# The overlap between Alzheimer's disease and epilepsy uncovered by transcriptome sequencing

**DOI:** 10.1002/ctm2.169

**Published:** 2020-09-11

**Authors:** Xiaowen Jiang, Hongyuan Lu, Wenwu Liu, Zhongchan Wu, Qiong Wu, Xiang Li, Zihua Xu, Fuhai Hui, Qingchun Zhao

**Affiliations:** ^1^ Department of Clinical Pharmacy Shenyang Pharmaceutical University Shenyang P. R. China; ^2^ Department of Clinical Pharmacology China Medical University Shenyang P. R. China; ^3^ Department of Pharmacy General Hospital of Northern Theater Command Shenyang P. R. China

Dear editor,

We conducted a combinated analysis of the hippocampal transcriptome of Alzheimer's disease (AD) and epilepsy mice for the first time. Our study found that TNF‐α‐HIF‐1‐NF‐κB pathway axis and circadian rhythm pathway are involved in the pathogenesis of AD and epilepsy. Most importantly, *FZD7* is remarkable upregulated in the hippocampus of APP/PS1 mice and the temporal cortex of humans, which suggests that *FZD7* maybe an important target in the early pathological process of AD.

It is believed that AD and epilepsy are two distinct neurological diseases based on its main symptoms. More and more clinical data show that there is interaction between AD and epilepsy.^1^ According to a retrospective study of medical records of new‐onset unexplained epilepsy and myoclonus, the incidence rate of epilepsy is 13.4% for late‐onset AD patients (n = 1320).^2^ In addition, In a 4‐year follow‐up study in Taiwan, 4.7% of the 20 000 AD patients showed seizure symptoms.^3^ These are only clinical data, and the number of subclinical epilepsy‐like symptoms may be higher.

It is recognized that Down syndrome (DS) may be accompanied by seizures, and DS patients have typical neuropathological changes in AD,^4^ which further illustrates the overlap between epilepsy and AD in pathology. Although there are clinical and pathological commonality between epilepsy and AD, it is still unclear how and why epilepsy is associated with an increase in AD pathology. Our previous research found that the circadian rhythms pathway was significantly down‐regulated in the hippocampal CA3 region of AD and epilepsy patient.^5^ In this study, we attempt to investigate the core pathways and genes of epilepsy and AD.

We analyzed the differentially expressed genes (DEGs) in APP/PS1 transgenic mice (n = 3) and pentylenetetrazole (PTZ) kindled epileptiform mice (n = 3) versus C57B6/J mice (WT mice, n = 3) by transcriptome sequencing. As shown in Figure 1A, the volcano map showed a total of 2184 DEGs were identified from APP/PS1 mice compared with WT mice, with 1293 genes were upregulated. Meanwhile, 2574 DEGs were identified from epileptiform mice with 1447 genes were upregulated. The relationship between the three groups of DEGs was intuitively shown by the Wayne diagram (Figure S1A). DEGs clustering analysis illustrated that the proportion of AD upregulated genes was more than that of epilepsy mice (Figure S1B).

**FIGURE 1:**
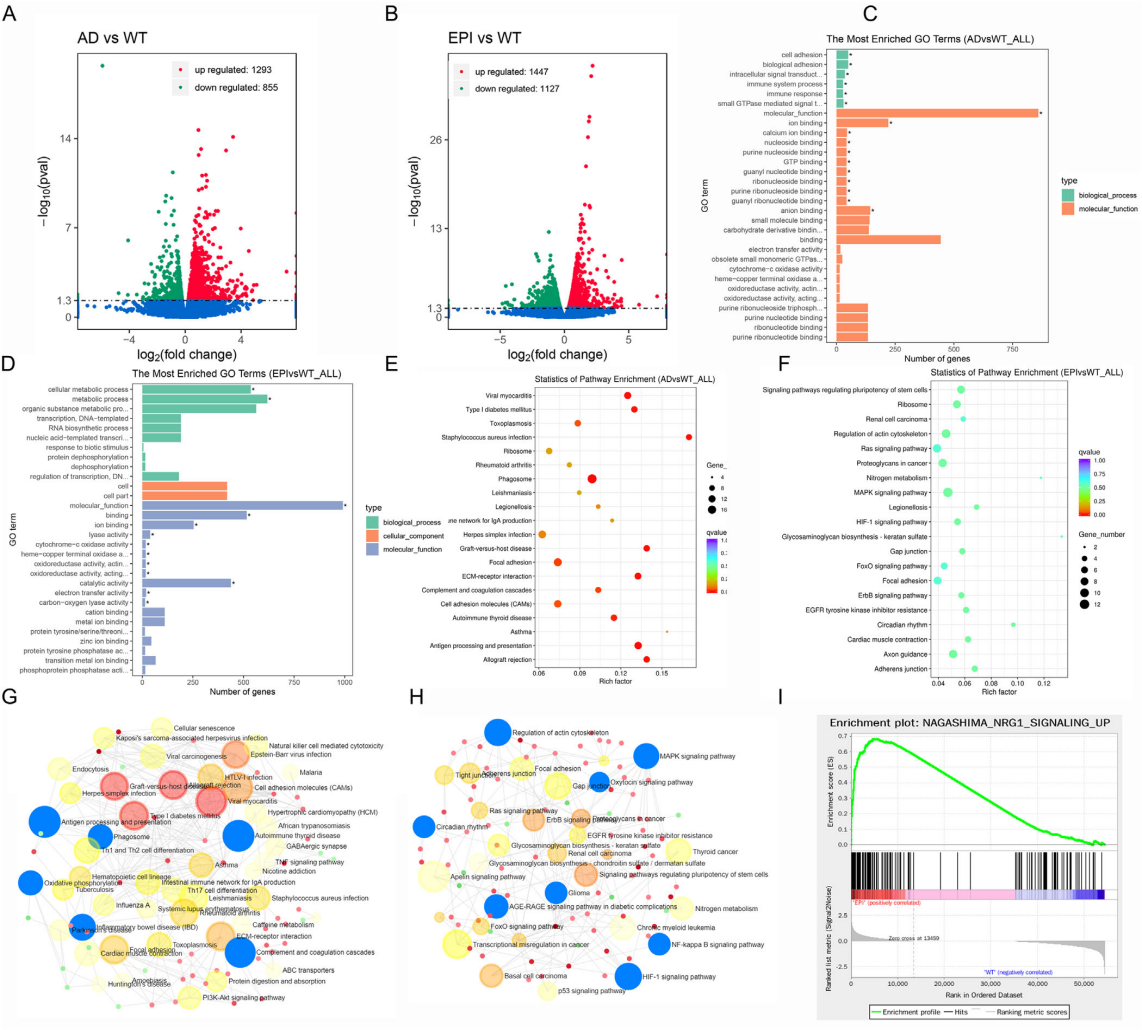
Analysis of physiological function signal pathway of DEGs. Volcano plot of the DEGs in APP/PS1 mice (A) and epileptiform mice (B). GO enrichment of DEGs in APP/PS1 mice (C) and epileptiform mice (D). KEGG pathways enrichment in APP/PS1 mice (E) and epileptiform mice (F). PPI pathway network analysis in APP/PS1 mice (G) and epileptiform mice (H). I, Enrichment plot of Nagashima_ NRG1_ SIGNALING_ Up in epileptiform mice by GSEA analysis

Figure 1C showed that the most enriched upregulated GO terms in AD mice were cell adhesion, biological adhesin, intracellular signal transduction, immune system process, and immune response. While, cellular metabolic process and metabolic process were the most enriched up‐regulated GO terms in epileptic mice (Figure 1D). In order to determine the signal transduction pathways involved in DEGs,^6^ we conducted KEGG analysis. Figure 1E illustrated that phagosome (corrected *P *= 0.0014) and antigen processing and presentation (corrected *P *= 0.0014) pathways were significant up‐regulated in APP/PS1 mice. The circadian rhythm pathway was downregulated in epileptic mice (Figure 1F), which verified our previous research.^5^

In order to analyze protein protein interaction (PPI), we input two sets of DEGs into NetworkAnalyst 3.0,^7^ and the results are similar to KEGG analysis (Figure 1G,H). See the Supporting Information Materials for details. Through the analysis of the pathway, we found that Alzheimer's disease pathway was significantly enriched in both AD and epileptic mice (Figure S2A). Next, we further analyzed the enrichment pathway of AD and epileptic mice with GSEA. In GSEA analysis, 26 gene sets were significant at FDR < 25% in epileptic mice. We were surprised to find the gene set, Nagashima_ NRG1_ SIGNALING_ Up, was the most significant enriched phenotype in epileptic mice, its NES = 2.16, FDR *q*‐val = 0 (Figure 1I). Neuregulin‐1 (NRG1) is a member of neurotrophic factors in the central nervous system (CNS). It is closely related to normal physiological functions such as neuronal growth, migration and differentiation, and synaptic plasticity via activating ErbBs receptors.^8^ NRG1 is also important for the translation of dopaminergic, glutamatergic, and GABAergic neurotransmitters.^9^ Our results suggest that NRG1 pathway may be a bridge between AD and epilepsy. In addition, Figure S2B showed that PHONG_TNF_TARGETS_UP was enriched in epileptiform mice with NES = 1.92, FDR *q*‐val = 0.05. Combined with the analysis results of HIF and NF‐κB pathway enriched in previous PPI analysis, it shows that TNF‐α‐HIF‐1‐NF‐κB pathway axis should be focused on in the pharmacological intervention mechanism of epilepsy and AD.

As illustrated in Figure 2A,B, PPI analysis of AD and epileptic mice mainly focus on neurodegenerative diseases pathways. As there are few common differential genes, we input these genes into the human AD database (Alzdata)^10^ to investigate their expression levels in human samples. We unexpectedly found that the expression of *FZD7* in the temporal cortex of AD patients was extremely upregulated than that of healthy people (*P* = 0.00017). Moreover, *FZD7* level was also significantly higher in entorhinal cortex than that of healthy people (*P* = 0.012; Figure 2C). Using brain single cell sequencing data in public databases (GSE67835), we found that *FZD7* is mainly distributed in astrocytes and neurons (Figure 2D). In the single gene analysis of *FZD7*, we conducted a network analysis of *FZD7*’s PPI, acting genes, related lncRNA, target miRNA, and indirectly acting genes (Figure 2E). Figure 2F is the research route of this study and the mechanism summary of FZD7. As a G‐protein coupled receptors, there are few studies on FZD7 that mainly focusing on cancer at present. In this study, we found for the first time that *FZD7* is remarkable upregulated in temporal cortex in AD patients. As an important part of cognition, temporal cortex is very vulnerable to attacks in the early stages of AD. These results suggest that the inhibition of *FZD7* in the early stages may prevent the progression of AD pathology and further systematic biological experiments are needed to verify it.

**FIGURE 2:**
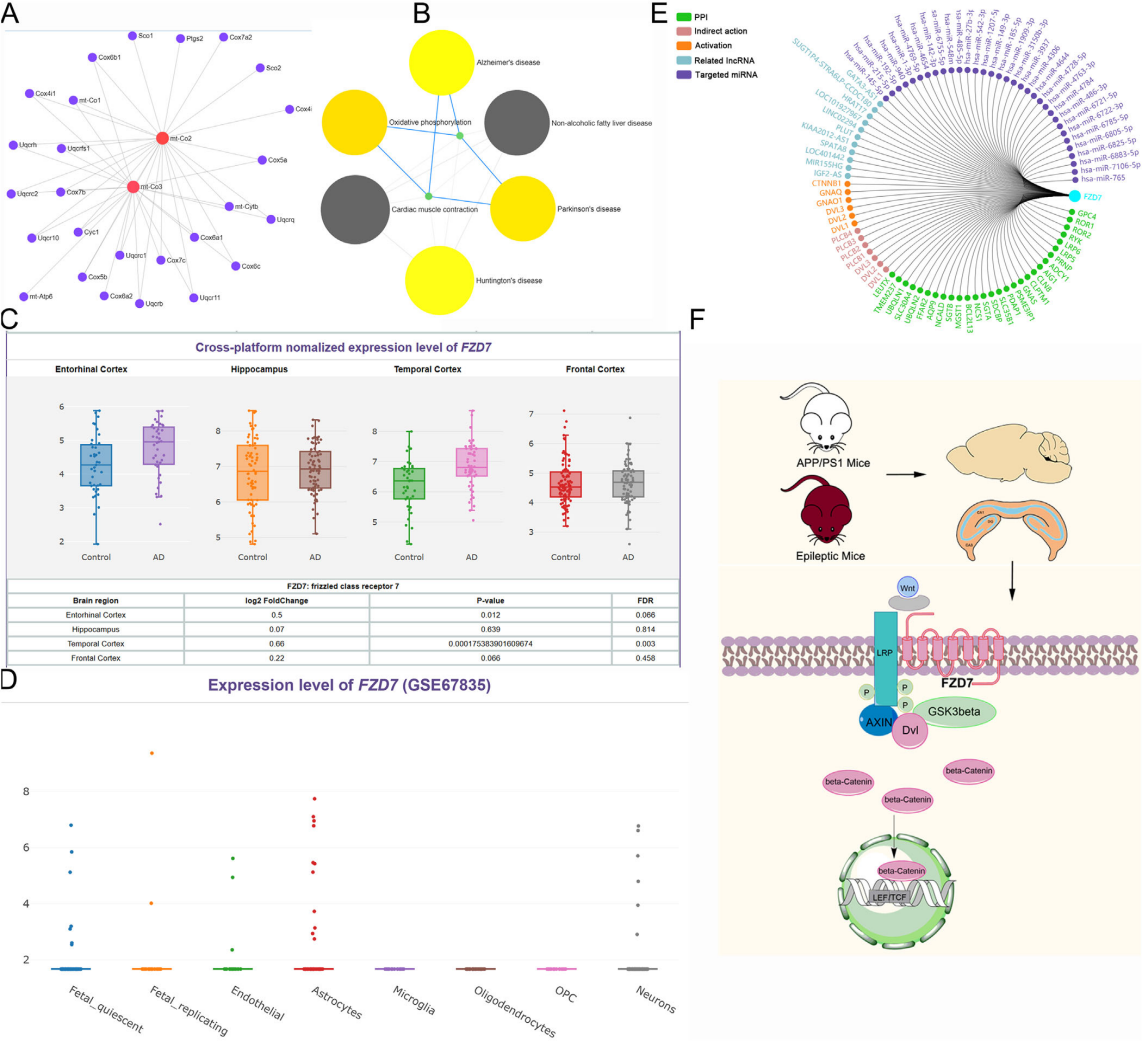
Functional analysis of core genes of AD and epilepsy. A, PPI gene network of APP/PS1 mice and epilepsy mice. B, PPI pathway network of APP/PS1 mice and epilepsy mice. C, *FZD7* level in human sample. D, The distribution of *FZD7* in brain single cell sequencing data (GSE67835). E, Gene regulatory network of *FZD7*. F, Research route and the mechanism summary of FZD7

## AUTHOR CONTRIBUTIONS

Qingchun Zhao and Fuhai Hui designed and supervised this study. Xiaowen Jiang performed experiments and bioinformatics analysis. Hongyuan Lu collected data and ran the analyses. Wenwu Liu, Zhongchan Wu, Qiong Wu, Xiang Li, and Zihua Xu discussed and commented on the manuscript.

## ETHICS APPROVAL AND CONSENT TO PARTICIPATE

All animal experiments were carried out in accordance with the National Institutes of Health guide for the care and use of laboratory animals (NIH Publications No. 8023, revised 1978). This study was approved by the Ethics Committee of the Institutional Animal Care and Use Committee of Shenyang Pharmaceutical University.

## CONFLICT OF INTEREST

The authors declare no conflict of interest.

## Supporting information

Supporting Information

Supporting Information

Supporting Information

Supporting Information

## Data Availability

The data that support the findings of the current study are available from the corresponding author on reasonable request.
