# Supplementary material for: The overlap between Alzheimer's disease and epilepsy uncovered by transcriptome sequencing
Source: Clin Transl Med. 2020 Sep 11;10(5):e169. doi: 10.1002/ctm2.169 (PMC7507445; doi:10.1002/ctm2.169)
Supplement: Supplementary file 1 — Supporting Information [file CTM2-10-e169-s001.docx]

**The overlap between Alzheimer’s disease and epilepsy uncovered by transcriptome sequencing**


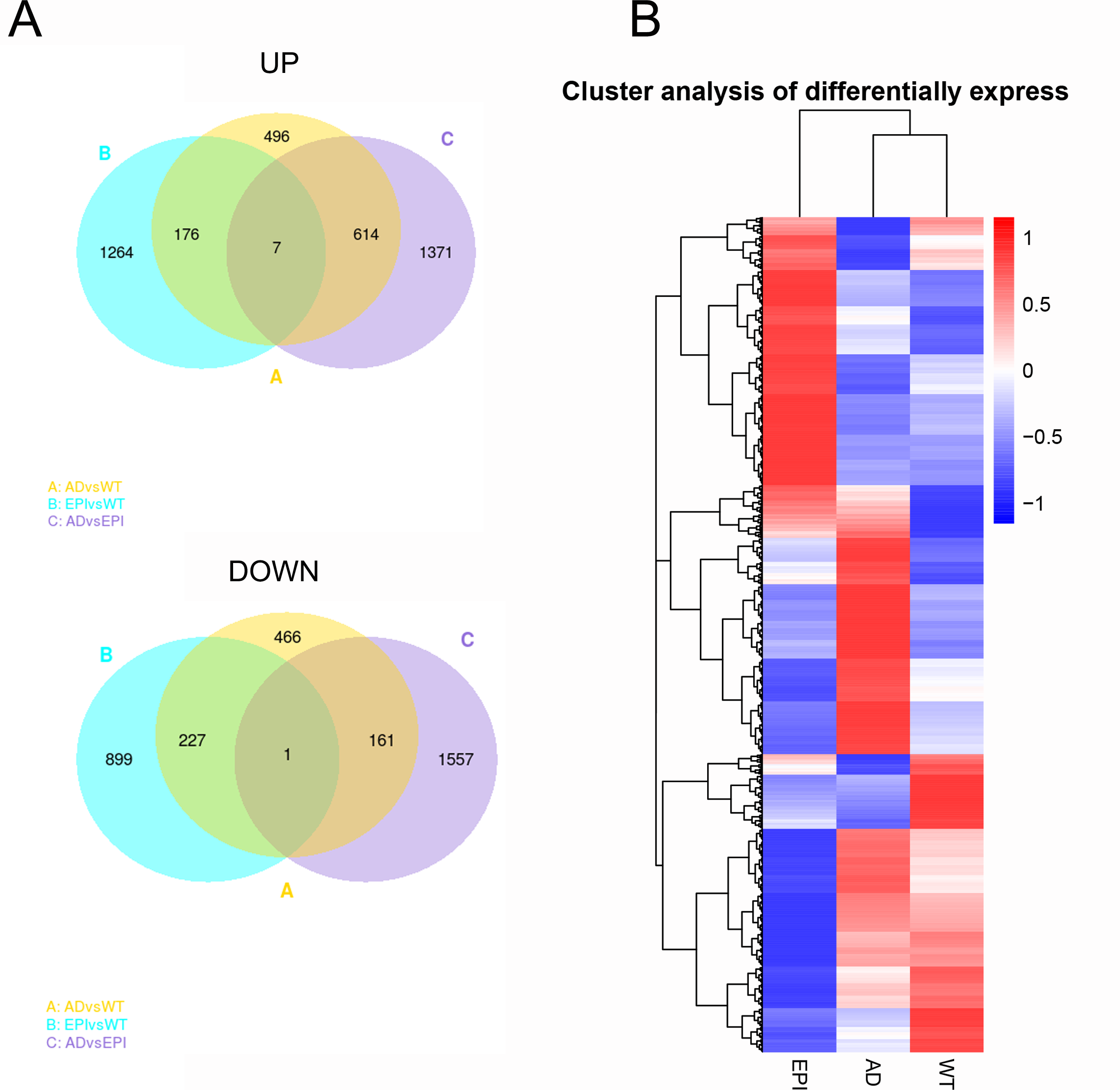


**Figure S1** A, Venn diagrams of the DEGs in APP/PS1 mice and epileptiform mice. B, Heatmap of the DEGs in APP/PS1 mice and epileptiform mice.


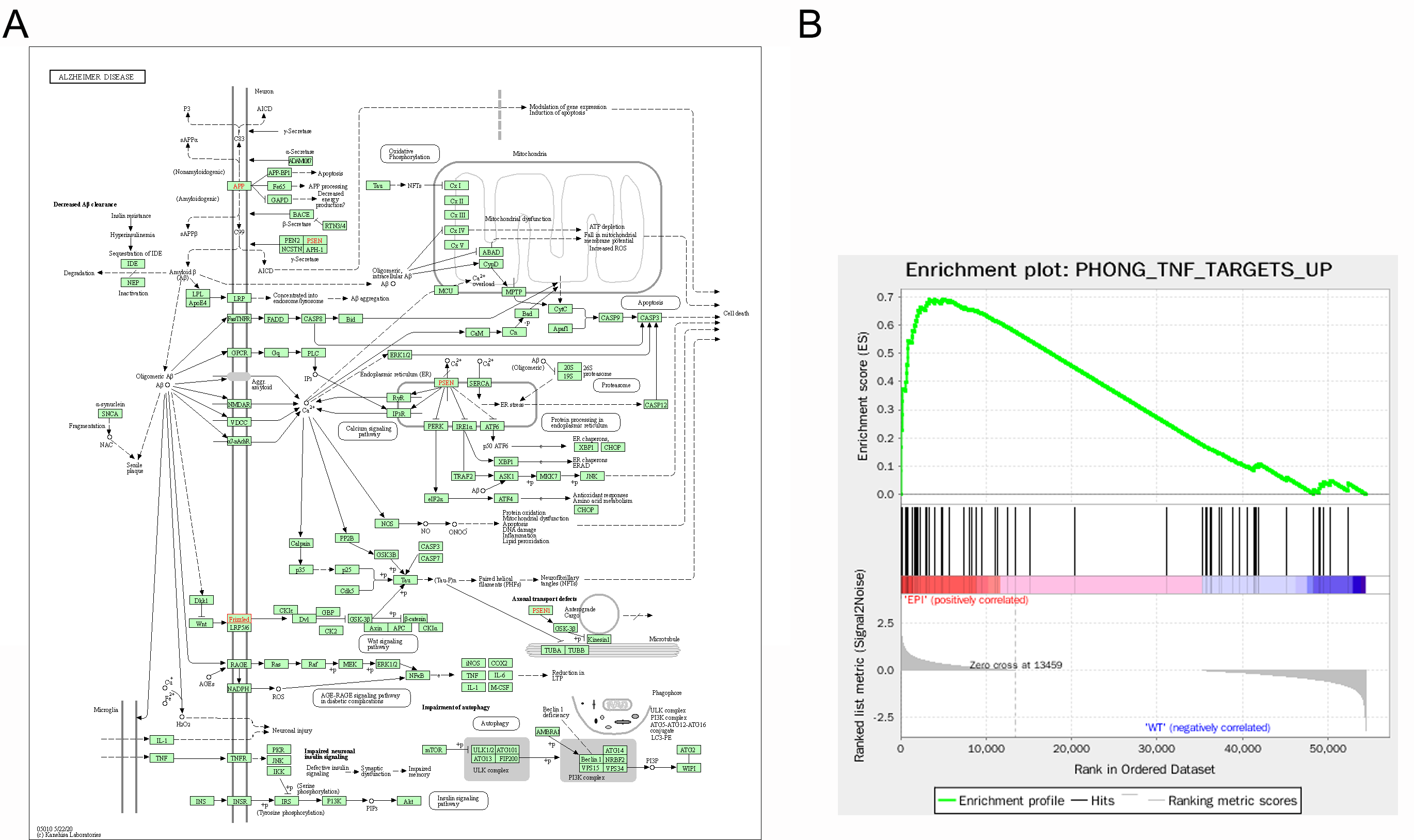


**Figure S2** A, Alzheimer’s disease pathway enriched both in APP/PS1 mice and epileptiform mice. B, Enrichment plot of PHONG_TNF_TARGETS_UP in epileptiform mice by GSEA analysis.
