# Supplementary material for: The overlap between Alzheimer's disease and epilepsy uncovered by transcriptome sequencing
Source: Clin Transl Med. 2020 Sep 11;10(5):e169. doi: 10.1002/ctm2.169 (PMC7507445; doi:10.1002/ctm2.169)
Supplement: Supplementary file 2 — Supporting Information [file CTM2-10-e169-s002.pdf]

20200710104259478916176178475008.docx

WORD COUNT	927	TIME SUBMITTED	10-JUL-2020 04:48AM
		PAPER ID	61038018

We conducted a joint analysis of the hippocampal transcriptome of AD and epilepsy mice for the first time. Our study found that TNF-α-HIF-1-NF-κB pathway axis and circadian rhythm pathway are involved in the pathogenesis of AD and epilepsy. Most importantly, *FZD7* is remarkable up-regulated in the hippocampus of APP/PS1 mice and the temporal cortex of human which suggests that *FZD7* is an important potential target in the early pathological process of AD.

It is believed that Alzheimer's disease (AD) and epilepsy are two distinct neurological diseases based on its main symptoms. More and more clinical data show that there is interaction between AD and epilepsy.[1] According to a retrospective study of medical records of new-onset unexplained epilepsy and myoclonus, the incidence rate of epilepsy is 13.4% for late-onset AD patients (n=1320).[2] In addition, In a 4-year follow-up study in Taiwan, 4.7% of the 20,000 AD patients showed seizure symptoms.[3] These are only clinical data, and the number of subclinical epilepsy-like symptoms may be higher.

It is recognized that Down syndrome (DS) may be accompanied by seizures, and DS patients have typical neuropathological changes in AD[4], which further illustrates the overlap between epilepsy and AD in pathology. Although there are clinical and pathological commonality between epilepsy and AD, it is still unclear how and why epilepsy is associated with an increase in AD pathology. Our previous research found that the circadian rhythms pathway was significantly down-regulated in the hippocampal CA3 region of AD and epilepsy patient.[5] In this study, we attempt to investigate the core pathways and key genes of epilepsy and AD.

We analyzed the differentially expressed genes (DEGs) in APP/PS1 transgenic mice and pentylenetetrazole (PTZ) induced epileptiform mice vs C57B6/J mice (WT mice) by transcriptome sequencing. As shown in Figure 1A, the volcano map showed a total of 2184 DEGs were identified from APP/PS1 mice compared with WT mice, with 1293 genes were up-regulated. Meanwhile, 2574 DEGs were identified from epileptiform mice with 1447 genes were up-regulated. The relationship between the three groups of DEGs was intuitively shown by the Wayne diagram (Figure S1A). DEGs clustering analysis illustrated that the proportion of AD up-regulated genes was more than that of

epilepsy mice (Figure S1B).

Figure 1C showed that the most enriched up-regulated GO terms in AD mice were cell adhesion, biological adhesin, intracellular signal transduction, immune system process, and immune response. While, cellular metabolic process and metabolic process were the most enriched up-regulated GO terms in epileptic mice (Figure 1D). In order to determine the signal transduction pathways involved in DEGs[6], we conducted KEGG analysis. Figure 1E illustrated that phagosome (corrected P=0.0014) and antigen processing and presentation (corrected P=0.0014) pathways were significant up-regulated in APP/PS1 mice. The circadian rhythm pathway was down-regulated in epileptic mice (Figure 1F) which verified our previous research.

In order to analyze protein protein interaction (PPI), we input two sets of DEGs into NetworkAnalyst 3.0,[7] and the results are similar to KEGG analysis (Figure 1G and H). See the supplementary materials for details. Through the analysis of the pathway, we found that Alzheimer's disease pathway was significantly enriched in both AD and epileptic mice (Figure S2A). Next, we further analyzed the enrichment pathway of AD and epileptic mice with GSEA. In GSEA analysis, 26 gene sets are significant at FDR<25% in epileptic mice. We were surprised to find the gene set, Nagashima_ NRG1_ SIGNALING_ Up, was the most significant enriched phenotype in epileptic mice, its NES=2.16, FDR q-val=0 (Figure 2I). Neuregulin-1 (NRG1) is a member of many neurotrophic factors in the central nervous system (CNS). It is closely related to normal physiological functions such as neuronal growth, migration and differentiation, and synaptic plasticity via activating ErbBs receptors.[8] NRG1 is also important for the translation of dopaminergic, glutamatergic and GABAergic neurotransmitters.[9] Our results suggest that NRG1 pathway may be a bridge between AD and epilepsy, and may be a potential target for AD drug therapy. In addition, Figure S2B showed that PHONG_TNF_TARGETS_UP was enriched in epileptiform mice with NES=1.92, FDR q-val=0.05. Combined with the analysis results of HIF and NF-κB pathway enriched in previous PPI analysis, it shows that TNF-α-HIF-1-NF-κB pathway axis should be focused on in the pharmacological intervention mechanism of epilepsy and AD.

As illustrated in Figure 2A and B, PPI analysis of AD and epileptic mice mainly focus on neurodegenerative diseases pathways. As there are few common differential genes, we input these genes into the human AD database (Alzdata)[10] to investigate their expression levels in human samples. We unexpectedly found that the expression of *FZD7* in the temporal cortex of AD patients was extremely upregulated than that of healthy people (*P*=0.00017). Moreover, *FZD7* level was also significantly higher in entorhinal cortex than that of healthy people (*P*=0.012) (Figure 2C). Using brain single cell sequencing data in public databases (GSE67835), we found that *FZD7* is mainly distributed in astrocytes and neurons (Figure 2D). In the single gene analysis of *FZD7*, we conducted a network analysis of *FZD7*'s PPI, acting genes, related lncRNA, target miRNA and indirectly acting genes (Figure 2E). Figure 2F is the research route of this study and the mechanism summary of FZD7. As a G protein coupled receptors, there are few studies on FZD7 which mainly focusing on cancer at present. In this study, we found for the first time that *FZD7* is remarkable up-regulated in temporal cortex in AD patients. As an important part of cognition, temporal cortex is very vulnerable to attacks in the early stages of AD. This suggests that the inhibition of *FZD7* in the early stages may prevent the progression of AD pathology.

20200710104259478916176178475008.docx

ORIGINALITY REPORT

0%

SIMILARITY INDEX

PRIMARY SOURCES

EXCLUDE QUOTES	OFF	EXCLUDE MATCHES	OFF
EXCLUDE BIBLIOGRAPHY	ON		
